# *N*-[5-(Di­phenyl­phosphorylmeth­yl)-4-(4-fluoro­phen­yl)-6-iso­propyl­pyrimi­din-2-yl]-*N*-methyl­methane­sulfonamide

**DOI:** 10.1107/S1600536813028286

**Published:** 2013-10-19

**Authors:** Ya-Ming Wu

**Affiliations:** a Department of Applied Chemistry, Nanjing College of Chemical Technology, No. 625 Geguan Road, Dachang, Nanjing 210048, People’s Republic of China

## Abstract

In the title compound, C_28_H_29_FN_3_O_3_PS, the pyrimidine ring is oriented at a dihedral angle of 50.9 (2)° with respect to the floro­benzene ring, while the two phenyl rings bonding to the same P atom are twisted with respect to each other, making a dihedral angle of 62.2 (2)°. In the crystal, mol­ecules are linked by weak C—H⋯O and C—H⋯F hydrogen bonds into a three-dimensional supra­molecular architecture.

## Related literature

For the synthesis of the title compound, an inter­mediate for the preparation of the statin rosuvastation {systematic name: (3*R*,5*S*,6*E*)-7-[4-(4-fluoro­phen­yl)-2-(*N*-methyl­methane­sulfon­amido)-6-(propan-2-yl)pyrimidin-5-yl]-3,5-di­hydroxy­hept-6-enoic acid}, see: Brieden & Veith (2000 ▶).
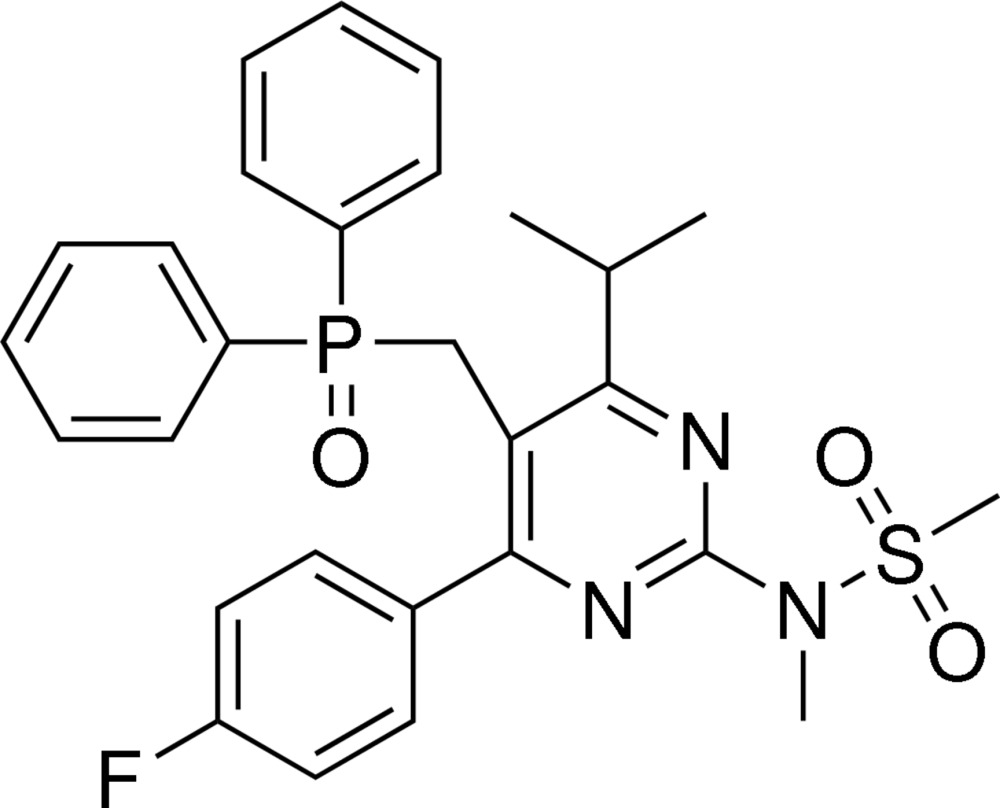


## Experimental

### 

#### Crystal data

C_28_H_29_FN_3_O_3_PS
*M* *_r_* = 537.57
Monoclinic, 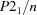

*a* = 14.023 (3) Å
*b* = 6.3830 (13) Å
*c* = 30.493 (6) Å
β = 102.79 (3)°
*V* = 2661.7 (9) Å^3^
*Z* = 4
Mo *K*α radiation
μ = 0.22 mm^−1^
*T* = 293 K
0.20 × 0.10 × 0.10 mm

#### Data collection

Enraf–Nonius CAD-4 diffractometer
Absorption correction: ψ scan (North *et al.*, 1968 ▶) *T*_min_ = 0.957, *T*_max_ = 0.978
5109 measured reflections
4898 independent reflections
2568 reflections with *I* > 2σ(*I*)
*R*_int_ = 0.092
3 standard reflections every 200 reflections intensity decay: 1%

#### Refinement

*R*[*F* ^2^ > 2σ(*F* ^2^)] = 0.065
*wR*(*F* ^2^) = 0.136
*S* = 1.00
4898 reflections
334 parameters
H-atom parameters constrained
Δρ_max_ = 0.29 e Å^−3^
Δρ_min_ = −0.26 e Å^−3^

### 

Data collection: *CAD-4 EXPRESS* (Enraf–Nonius, 1994 ▶); cell refinement: *CAD-4 EXPRESS*; data reduction: *XCAD4* (Harms & Wocadlo, 1995 ▶); program(s) used to solve structure: *SHELXTL* (Sheldrick, 2008 ▶); program(s) used to refine structure: *SHELXTL*; molecular graphics: *SHELXTL*; software used to prepare material for publication: *SHELXTL*.

## Supplementary Material

Crystal structure: contains datablock(s) I, New_Global_Publ_Block. DOI: 10.1107/S1600536813028286/xu5743sup1.cif

Structure factors: contains datablock(s) I. DOI: 10.1107/S1600536813028286/xu5743Isup2.hkl

Supplementary material file. DOI: 10.1107/S1600536813028286/xu5743Isup3.cml

Additional supplementary materials:  crystallographic information; 3D view; checkCIF report

## Figures and Tables

**Table 1:** Hydrogen-bond geometry (Å, °)

*D*—H⋯*A*	*D*—H	H⋯*A*	*D*⋯*A*	*D*—H⋯*A*
C1—H1*B*⋯O1^i^	0.96	2.56	3.363 (5)	141
C2—H2*C*⋯F^ii^	0.96	2.52	3.202 (6)	128
C13—H13*A*⋯O2^iii^	0.93	2.53	3.350 (6)	148

Symmetry codes: (i) 
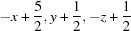
; (ii) 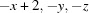; (iii) 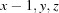.

**Fig. 1.**
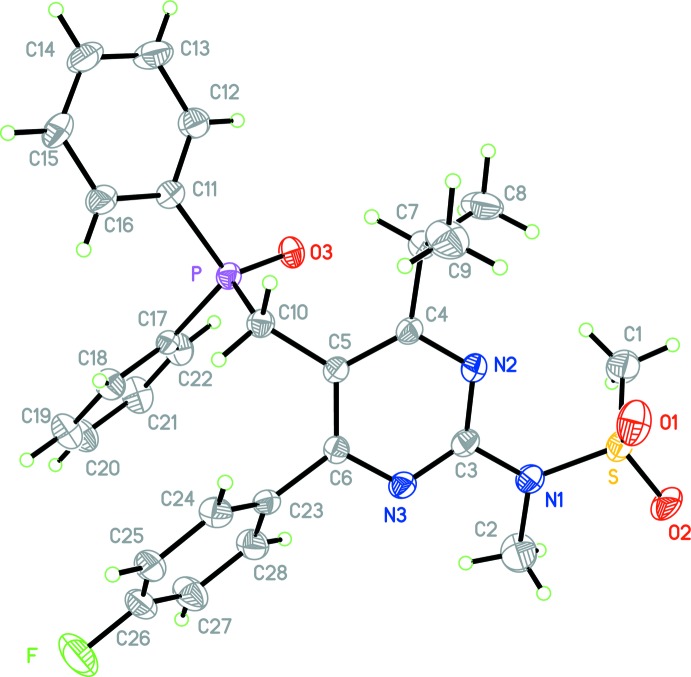
The molecular structure of (I), with the atom-numbering scheme. Displacement ellipsoids are drawn at the 50% probability level.

**Fig. 2.**
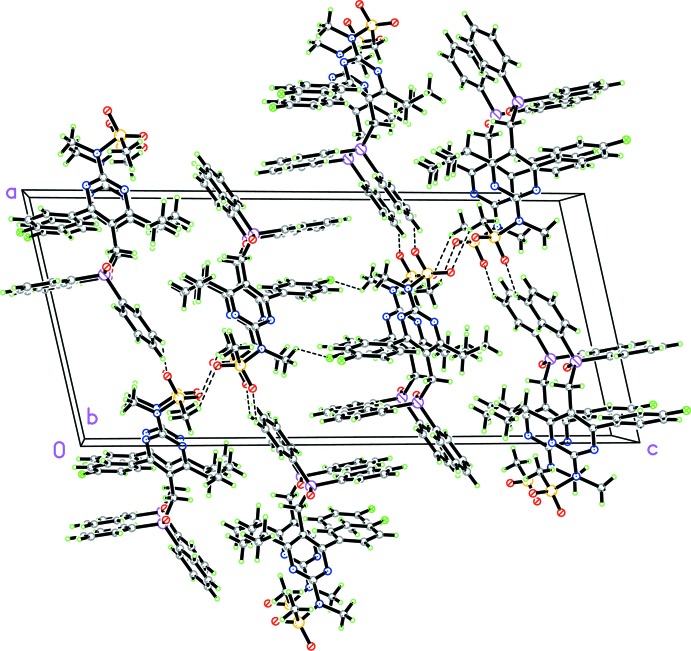
A packing diagram of (I).

## supplementary crystallographic information

### 1. Comment

The title compound is an intermediate for the preparation of rosuvastation (Brieden & Veith, 2000). We herein report its molecular and crystal structure (Fig. 1). The dihedral angles between the aromatic rings are: 30.7 (2)° (A/B), 65.76 (1)° (A/D), 50.9 (2)°(A/E), and 62.2 (2)° (B/D) [with the rings defined as: A =C3/N2/C4—C6/N3, B=C11—C16, D=C17—C22 and E=C23—C28]. In the crystal structure, no classic hydrogen bond was observed and molecules were stacked to form three-dimensional framework by weak C—H···O and C—H···F interactions (Table 1) (Fig. 2).

### 2. Experimental

The title compound was synthesized according to a procedure published by Brieden & Veith (2000). Colorless crystals suitable for X-ray analysis were obtained by dissolving the compound (0.5 g) in ethanol (80 ml) and evaporating the solvent slowly at room temperature for about 5 d.

### 3. Refinement

H atoms were positioned geometrically with C—H = 0.93-0.98 Å and constrained to ride on their parent atoms, U_iso_(H) = 1.5U_eq_(C) for methyl H atoms and 1.2U_eq_(C) for the others.

### Crystal data

**Table:**

C_28_H_29_FN_3_O_3_PS	*F*(000) = 1128
*M**_r_* = 537.57	*D*_x_ = 1.342 Mg m^−^^3^
Monoclinic, *P*2_1_/*n*	Mo *K*α radiation, λ = 0.71073 Å
Hall symbol: -P 2yn	Cell parameters from 25 reflections
*a* = 14.023 (3) Å	θ = 9–13°
*b* = 6.3830 (13) Å	µ = 0.22 mm^−^^1^
*c* = 30.493 (6) Å	*T* = 293 K
β = 102.79 (3)°	Block, colorless
*V* = 2661.7 (9) Å^3^	0.20 × 0.10 × 0.10 mm
*Z* = 4	

### Data collection

**Table:**

Enraf–Nonius CAD-4 diffractometer	2568 reflections with *I* > 2σ(*I*)
Radiation source: fine-focus sealed tube	*R*_int_ = 0.092
Graphite monochromator	θ_max_ = 25.5°, θ_min_ = 1.4°
ω/2θ scans	*h* = 0→16
Absorption correction: ψ scan (North *et al.*, 1968)	*k* = 0→7
*T*_min_ = 0.957, *T*_max_ = 0.978	*l* = −36→35
5109 measured reflections	3 standard reflections every 200 reflections
4898 independent reflections	intensity decay: 1%

### Refinement

**Table:**

Refinement on *F*^2^	Primary atom site location: structure-invariant direct methods
Least-squares matrix: full	Secondary atom site location: difference Fourier map
*R*[*F*^2^ > 2σ(*F*^2^)] = 0.065	Hydrogen site location: inferred from neighbouring sites
*wR*(*F*^2^) = 0.136	H-atom parameters constrained
*S* = 1.00	*w* = 1/[σ^2^(*F*_o_^2^) + (0.0378*P*)^2^] where *P* = (*F*_o_^2^ + 2*F*_c_^2^)/3
4898 reflections	(Δ/σ)_max_ < 0.001
334 parameters	Δρ_max_ = 0.29 e Å^−^^3^
0 restraints	Δρ_min_ = −0.26 e Å^−^^3^

### Special details

**Table:**

Geometry. All e.s.d.'s (except the e.s.d. in the dihedral angle between two l.s. planes) are estimated using the full covariance matrix. The cell e.s.d.'s are taken into account individually in the estimation of e.s.d.'s in distances, angles and torsion angles; correlations between e.s.d.'s in cell parameters are only used when they are defined by crystal symmetry. An approximate (isotropic) treatment of cell e.s.d.'s is used for estimating e.s.d.'s involving l.s. planes.
Refinement. Refinement of *F*^2^ against ALL reflections. The weighted *R*-factor *wR* and goodness of fit *S* are based on *F*^2^, conventional *R*-factors *R* are based on *F*, with *F* set to zero for negative *F*^2^. The threshold expression of *F*^2^ > σ(*F*^2^) is used only for calculating *R*-factors(gt) *etc*. and is not relevant to the choice of reflections for refinement. *R*-factors based on *F*^2^ are statistically about twice as large as those based on *F*, and *R*- factors based on ALL data will be even larger.

### Fractional atomic coordinates and isotropic or equivalent isotropic displacement parameters (Å^2^)

**Table:**

	*x*	*y*	*z*	*U*_iso_*/*U*_eq_	
P	0.66722 (7)	0.42516 (17)	0.11070 (4)	0.0362 (3)	
S	1.18096 (8)	0.76834 (19)	0.19108 (4)	0.0487 (3)	
F	0.8413 (2)	−0.3088 (5)	−0.02311 (10)	0.0980 (11)	
O1	1.1845 (2)	0.6427 (5)	0.22969 (10)	0.0686 (10)	
N1	1.1193 (2)	0.6413 (5)	0.14580 (11)	0.0482 (9)	
C1	1.1139 (3)	0.9950 (7)	0.19474 (16)	0.0659 (14)	
H1B	1.1459	1.0751	0.2204	0.099*	
H1C	1.0494	0.9576	0.1978	0.099*	
H1D	1.1094	1.0771	0.1680	0.099*	
N2	0.9853 (2)	0.5871 (5)	0.17771 (10)	0.0398 (8)	
O2	1.2701 (2)	0.8296 (5)	0.17934 (11)	0.0700 (10)	
C2	1.1635 (4)	0.6142 (9)	0.10687 (15)	0.0852 (18)	
H2C	1.1191	0.5392	0.0837	0.128*	
H2D	1.2233	0.5365	0.1157	0.128*	
H2E	1.1771	0.7490	0.0957	0.128*	
O3	0.69197 (19)	0.6503 (4)	0.11812 (9)	0.0459 (7)	
N3	1.0068 (2)	0.3895 (5)	0.11488 (11)	0.0397 (8)	
C3	1.0327 (3)	0.5313 (6)	0.14613 (14)	0.0399 (10)	
C4	0.9008 (3)	0.4874 (6)	0.17684 (13)	0.0361 (10)	
C5	0.8634 (3)	0.3368 (6)	0.14377 (12)	0.0339 (9)	
C6	0.9213 (3)	0.2883 (6)	0.11349 (12)	0.0341 (9)	
C7	0.8527 (3)	0.5433 (7)	0.21492 (13)	0.0439 (11)	
H7A	0.7824	0.5165	0.2050	0.053*	
C8	0.8664 (4)	0.7705 (7)	0.22873 (16)	0.0714 (15)	
H8A	0.8392	0.8583	0.2035	0.107*	
H8B	0.9350	0.8000	0.2387	0.107*	
H8C	0.8340	0.7977	0.2527	0.107*	
C9	0.8930 (4)	0.3984 (8)	0.25420 (14)	0.0732 (16)	
H9A	0.8824	0.2553	0.2446	0.110*	
H9B	0.8601	0.4251	0.2781	0.110*	
H9C	0.9618	0.4230	0.2647	0.110*	
C10	0.7621 (2)	0.2477 (6)	0.14028 (12)	0.0378 (10)	
H10A	0.7520	0.2215	0.1703	0.045*	
H10B	0.7569	0.1149	0.1245	0.045*	
C11	0.5601 (3)	0.3550 (6)	0.13092 (13)	0.0369 (10)	
C12	0.5178 (3)	0.5088 (7)	0.15180 (14)	0.0511 (12)	
H12A	0.5440	0.6432	0.1542	0.061*	
C13	0.4366 (3)	0.4668 (8)	0.16934 (16)	0.0672 (15)	
H13A	0.4086	0.5722	0.1834	0.081*	
C14	0.3980 (3)	0.2691 (9)	0.16577 (16)	0.0622 (14)	
H14A	0.3441	0.2395	0.1778	0.075*	
C15	0.4384 (3)	0.1138 (8)	0.14443 (15)	0.0628 (14)	
H15A	0.4108	−0.0193	0.1415	0.075*	
C16	0.5201 (3)	0.1545 (7)	0.12736 (14)	0.0513 (12)	
H16A	0.5482	0.0484	0.1135	0.062*	
C17	0.6458 (3)	0.3547 (6)	0.05202 (13)	0.0346 (9)	
C18	0.6367 (3)	0.1505 (7)	0.03646 (14)	0.0485 (11)	
H18A	0.6428	0.0402	0.0568	0.058*	
C19	0.6186 (3)	0.1096 (7)	−0.00913 (15)	0.0580 (13)	
H19A	0.6121	−0.0280	−0.0194	0.070*	
C20	0.6103 (3)	0.2717 (8)	−0.03926 (15)	0.0582 (13)	
H20A	0.5986	0.2443	−0.0699	0.070*	
C21	0.6194 (3)	0.4743 (8)	−0.02397 (15)	0.0603 (13)	
H21A	0.6144	0.5841	−0.0444	0.072*	
C22	0.6357 (3)	0.5165 (7)	0.02123 (15)	0.0512 (12)	
H22A	0.6401	0.6546	0.0312	0.061*	
C23	0.8973 (3)	0.1289 (6)	0.07731 (14)	0.0376 (10)	
C24	0.8742 (3)	−0.0748 (6)	0.08606 (14)	0.0451 (11)	
H24A	0.8713	−0.1126	0.1152	0.054*	
C25	0.8553 (3)	−0.2243 (7)	0.05210 (16)	0.0528 (12)	
H25A	0.8401	−0.3618	0.0580	0.063*	
C26	0.8598 (3)	−0.1626 (8)	0.00999 (17)	0.0594 (13)	
C27	0.8826 (4)	0.0346 (8)	−0.00001 (16)	0.0632 (14)	
H27A	0.8848	0.0706	−0.0293	0.076*	
C28	0.9028 (3)	0.1825 (7)	0.03403 (16)	0.0545 (12)	
H28A	0.9200	0.3181	0.0277	0.065*	

### Atomic displacement parameters (Å^2^)

**Table:**

	*U*^11^	*U*^22^	*U*^33^	*U*^12^	*U*^13^	*U*^23^
P	0.0343 (6)	0.0324 (6)	0.0427 (7)	−0.0038 (5)	0.0105 (5)	−0.0012 (5)
S	0.0338 (6)	0.0486 (7)	0.0621 (8)	−0.0049 (5)	0.0072 (5)	−0.0026 (6)
F	0.137 (3)	0.075 (2)	0.091 (2)	−0.028 (2)	0.044 (2)	−0.0440 (19)
O1	0.063 (2)	0.074 (2)	0.060 (2)	−0.0055 (18)	−0.0063 (16)	0.0205 (19)
N1	0.043 (2)	0.052 (2)	0.055 (2)	−0.0140 (18)	0.0212 (17)	−0.008 (2)
C1	0.054 (3)	0.050 (3)	0.091 (4)	0.008 (3)	0.011 (3)	−0.011 (3)
N2	0.0345 (19)	0.042 (2)	0.043 (2)	−0.0065 (17)	0.0085 (16)	−0.0027 (17)
O2	0.0371 (17)	0.066 (2)	0.113 (3)	−0.0235 (17)	0.0294 (17)	−0.023 (2)
C2	0.084 (4)	0.117 (5)	0.068 (4)	−0.044 (4)	0.045 (3)	−0.015 (4)
O3	0.0514 (17)	0.0305 (16)	0.0556 (19)	−0.0095 (14)	0.0112 (14)	−0.0055 (15)
N3	0.0356 (18)	0.037 (2)	0.049 (2)	−0.0030 (16)	0.0144 (16)	−0.0024 (18)
C3	0.035 (2)	0.041 (3)	0.045 (3)	−0.003 (2)	0.013 (2)	0.003 (2)
C4	0.030 (2)	0.040 (2)	0.038 (2)	0.0020 (19)	0.0060 (18)	0.002 (2)
C5	0.030 (2)	0.034 (2)	0.037 (2)	−0.0008 (19)	0.0076 (17)	0.001 (2)
C6	0.034 (2)	0.025 (2)	0.041 (2)	0.0005 (18)	0.0052 (18)	0.0013 (19)
C7	0.039 (2)	0.053 (3)	0.040 (2)	−0.003 (2)	0.010 (2)	−0.005 (2)
C8	0.085 (4)	0.060 (3)	0.083 (4)	0.004 (3)	0.048 (3)	−0.017 (3)
C9	0.091 (4)	0.088 (4)	0.044 (3)	−0.001 (3)	0.022 (3)	0.007 (3)
C10	0.038 (2)	0.036 (2)	0.040 (2)	−0.002 (2)	0.0104 (18)	0.001 (2)
C11	0.035 (2)	0.036 (2)	0.039 (2)	0.0038 (19)	0.0073 (18)	0.007 (2)
C12	0.054 (3)	0.043 (3)	0.060 (3)	0.000 (2)	0.019 (2)	−0.001 (2)
C13	0.056 (3)	0.071 (4)	0.085 (4)	0.010 (3)	0.037 (3)	−0.001 (3)
C14	0.041 (3)	0.081 (4)	0.071 (4)	−0.004 (3)	0.027 (2)	0.002 (3)
C15	0.049 (3)	0.065 (4)	0.081 (4)	−0.022 (3)	0.028 (3)	−0.003 (3)
C16	0.049 (3)	0.049 (3)	0.062 (3)	−0.008 (2)	0.026 (2)	−0.003 (2)
C17	0.030 (2)	0.033 (2)	0.041 (2)	−0.0037 (18)	0.0115 (18)	−0.005 (2)
C18	0.057 (3)	0.043 (3)	0.045 (3)	−0.003 (2)	0.013 (2)	0.001 (2)
C19	0.073 (3)	0.046 (3)	0.050 (3)	0.000 (3)	0.004 (2)	−0.007 (3)
C20	0.076 (3)	0.060 (3)	0.036 (3)	−0.002 (3)	0.007 (2)	−0.005 (3)
C21	0.080 (4)	0.053 (3)	0.044 (3)	−0.008 (3)	0.007 (3)	0.011 (3)
C22	0.060 (3)	0.035 (3)	0.056 (3)	−0.001 (2)	0.010 (2)	0.006 (2)
C23	0.033 (2)	0.037 (3)	0.045 (3)	0.0035 (19)	0.0113 (19)	−0.002 (2)
C24	0.053 (3)	0.037 (3)	0.048 (3)	0.005 (2)	0.015 (2)	0.005 (2)
C25	0.056 (3)	0.033 (3)	0.072 (3)	−0.002 (2)	0.019 (3)	−0.010 (3)
C26	0.070 (3)	0.047 (3)	0.069 (4)	−0.011 (3)	0.032 (3)	−0.023 (3)
C27	0.088 (4)	0.057 (3)	0.053 (3)	−0.012 (3)	0.031 (3)	−0.008 (3)
C28	0.064 (3)	0.038 (3)	0.068 (3)	−0.008 (2)	0.029 (3)	−0.005 (3)

### Geometric parameters (Å, º)

**Table:**

P—O3	1.484 (3)	C10—H10A	0.9700
P—C11	1.802 (4)	C10—H10B	0.9700
P—C17	1.805 (4)	C11—C12	1.374 (5)
P—C10	1.826 (4)	C11—C16	1.391 (5)
S—O1	1.416 (3)	C12—C13	1.387 (5)
S—O2	1.428 (3)	C12—H12A	0.9300
S—N1	1.668 (3)	C13—C14	1.368 (6)
S—C1	1.743 (4)	C13—H13A	0.9300
F—C26	1.357 (5)	C14—C15	1.375 (6)
N1—C3	1.404 (5)	C14—H14A	0.9300
N1—C2	1.467 (5)	C15—C16	1.384 (5)
C1—H1B	0.9600	C15—H15A	0.9300
C1—H1C	0.9600	C16—H16A	0.9300
C1—H1D	0.9600	C17—C22	1.381 (5)
N2—C3	1.334 (4)	C17—C18	1.383 (5)
N2—C4	1.341 (4)	C18—C19	1.382 (5)
C2—H2C	0.9600	C18—H18A	0.9300
C2—H2D	0.9600	C19—C20	1.372 (6)
C2—H2E	0.9600	C19—H19A	0.9300
N3—C3	1.306 (5)	C20—C21	1.371 (6)
N3—C6	1.354 (4)	C20—H20A	0.9300
C4—C5	1.408 (5)	C21—C22	1.373 (6)
C4—C7	1.509 (5)	C21—H21A	0.9300
C5—C6	1.393 (5)	C22—H22A	0.9300
C5—C10	1.512 (5)	C23—C24	1.380 (5)
C6—C23	1.484 (5)	C23—C28	1.382 (5)
C7—C8	1.511 (5)	C24—C25	1.390 (5)
C7—C9	1.520 (6)	C24—H24A	0.9300
C7—H7A	0.9800	C25—C26	1.358 (6)
C8—H8A	0.9600	C25—H25A	0.9300
C8—H8B	0.9600	C26—C27	1.350 (6)
C8—H8C	0.9600	C27—C28	1.385 (6)
C9—H9A	0.9600	C27—H27A	0.9300
C9—H9B	0.9600	C28—H28A	0.9300
C9—H9C	0.9600		
			
O3—P—C11	111.91 (18)	C5—C10—H10A	109.3
O3—P—C17	111.99 (18)	P—C10—H10A	109.3
C11—P—C17	107.99 (17)	C5—C10—H10B	109.3
O3—P—C10	113.90 (17)	P—C10—H10B	109.3
C11—P—C10	104.36 (17)	H10A—C10—H10B	107.9
C17—P—C10	106.17 (18)	C12—C11—C16	119.0 (4)
O1—S—O2	119.5 (2)	C12—C11—P	117.3 (3)
O1—S—N1	109.22 (19)	C16—C11—P	123.6 (3)
O2—S—N1	104.34 (18)	C11—C12—C13	121.1 (4)
O1—S—C1	109.7 (2)	C11—C12—H12A	119.5
O2—S—C1	107.7 (2)	C13—C12—H12A	119.5
N1—S—C1	105.6 (2)	C14—C13—C12	119.5 (4)
C3—N1—C2	117.9 (3)	C14—C13—H13A	120.3
C3—N1—S	121.9 (3)	C12—C13—H13A	120.3
C2—N1—S	119.5 (3)	C13—C14—C15	120.3 (4)
S—C1—H1B	109.5	C13—C14—H14A	119.8
S—C1—H1C	109.5	C15—C14—H14A	119.8
H1B—C1—H1C	109.5	C14—C15—C16	120.3 (4)
S—C1—H1D	109.5	C14—C15—H15A	119.8
H1B—C1—H1D	109.5	C16—C15—H15A	119.8
H1C—C1—H1D	109.5	C15—C16—C11	119.7 (4)
C3—N2—C4	116.0 (3)	C15—C16—H16A	120.1
N1—C2—H2C	109.5	C11—C16—H16A	120.1
N1—C2—H2D	109.5	C22—C17—C18	118.9 (4)
H2C—C2—H2D	109.5	C22—C17—P	117.2 (3)
N1—C2—H2E	109.5	C18—C17—P	123.9 (3)
H2C—C2—H2E	109.5	C19—C18—C17	120.4 (4)
H2D—C2—H2E	109.5	C19—C18—H18A	119.8
C3—N3—C6	117.0 (3)	C17—C18—H18A	119.8
N3—C3—N2	127.5 (4)	C20—C19—C18	120.1 (4)
N3—C3—N1	116.3 (3)	C20—C19—H19A	120.0
N2—C3—N1	116.2 (4)	C18—C19—H19A	120.0
N2—C4—C5	121.7 (3)	C21—C20—C19	119.7 (4)
N2—C4—C7	114.6 (3)	C21—C20—H20A	120.1
C5—C4—C7	123.6 (3)	C19—C20—H20A	120.1
C6—C5—C4	116.6 (3)	C20—C21—C22	120.6 (4)
C6—C5—C10	123.1 (3)	C20—C21—H21A	119.7
C4—C5—C10	120.1 (3)	C22—C21—H21A	119.7
N3—C6—C5	121.0 (3)	C21—C22—C17	120.3 (4)
N3—C6—C23	113.8 (3)	C21—C22—H22A	119.8
C5—C6—C23	125.2 (3)	C17—C22—H22A	119.8
C4—C7—C8	113.0 (3)	C24—C23—C28	118.9 (4)
C4—C7—C9	108.1 (3)	C24—C23—C6	121.8 (4)
C8—C7—C9	111.3 (4)	C28—C23—C6	119.3 (4)
C4—C7—H7A	108.1	C23—C24—C25	121.1 (4)
C8—C7—H7A	108.1	C23—C24—H24A	119.4
C9—C7—H7A	108.1	C25—C24—H24A	119.4
C7—C8—H8A	109.5	C26—C25—C24	117.6 (4)
C7—C8—H8B	109.5	C26—C25—H25A	121.2
H8A—C8—H8B	109.5	C24—C25—H25A	121.2
C7—C8—H8C	109.5	C27—C26—F	119.3 (5)
H8A—C8—H8C	109.5	C27—C26—C25	123.2 (4)
H8B—C8—H8C	109.5	F—C26—C25	117.5 (4)
C7—C9—H9A	109.5	C26—C27—C28	119.0 (4)
C7—C9—H9B	109.5	C26—C27—H27A	120.5
H9A—C9—H9B	109.5	C28—C27—H27A	120.5
C7—C9—H9C	109.5	C23—C28—C27	120.1 (4)
H9A—C9—H9C	109.5	C23—C28—H28A	119.9
H9B—C9—H9C	109.5	C27—C28—H28A	119.9
C5—C10—P	111.8 (3)		
			
O1—S—N1—C3	42.5 (4)	C17—P—C11—C16	55.1 (4)
O2—S—N1—C3	171.3 (3)	C10—P—C11—C16	−57.6 (4)
C1—S—N1—C3	−75.4 (4)	C16—C11—C12—C13	0.2 (6)
O1—S—N1—C2	−127.5 (4)	P—C11—C12—C13	−178.4 (3)
O2—S—N1—C2	1.3 (4)	C11—C12—C13—C14	0.0 (7)
C1—S—N1—C2	114.6 (4)	C12—C13—C14—C15	−0.9 (8)
C6—N3—C3—N2	1.1 (6)	C13—C14—C15—C16	1.6 (8)
C6—N3—C3—N1	−177.2 (3)	C14—C15—C16—C11	−1.5 (7)
C4—N2—C3—N3	−0.8 (6)	C12—C11—C16—C15	0.5 (6)
C4—N2—C3—N1	177.5 (3)	P—C11—C16—C15	179.0 (3)
C2—N1—C3—N3	10.0 (6)	O3—P—C17—C22	−12.2 (4)
S—N1—C3—N3	−160.2 (3)	C11—P—C17—C22	111.5 (3)
C2—N1—C3—N2	−168.5 (4)	C10—P—C17—C22	−137.1 (3)
S—N1—C3—N2	21.3 (5)	O3—P—C17—C18	169.4 (3)
C3—N2—C4—C5	−1.6 (5)	C11—P—C17—C18	−66.9 (4)
C3—N2—C4—C7	175.9 (3)	C10—P—C17—C18	44.5 (4)
N2—C4—C5—C6	3.4 (5)	C22—C17—C18—C19	0.5 (6)
C7—C4—C5—C6	−173.8 (4)	P—C17—C18—C19	178.9 (3)
N2—C4—C5—C10	−172.3 (3)	C17—C18—C19—C20	0.5 (7)
C7—C4—C5—C10	10.5 (6)	C18—C19—C20—C21	−0.4 (7)
C3—N3—C6—C5	1.0 (5)	C19—C20—C21—C22	−0.7 (7)
C3—N3—C6—C23	−179.6 (3)	C20—C21—C22—C17	1.7 (7)
C4—C5—C6—N3	−3.1 (5)	C18—C17—C22—C21	−1.6 (6)
C10—C5—C6—N3	172.5 (3)	P—C17—C22—C21	179.9 (3)
C4—C5—C6—C23	177.6 (3)	N3—C6—C23—C24	127.6 (4)
C10—C5—C6—C23	−6.8 (6)	C5—C6—C23—C24	−53.0 (6)
N2—C4—C7—C8	35.5 (5)	N3—C6—C23—C28	−49.2 (5)
C5—C4—C7—C8	−147.1 (4)	C5—C6—C23—C28	130.2 (4)
N2—C4—C7—C9	−88.1 (4)	C28—C23—C24—C25	−0.9 (6)
C5—C4—C7—C9	89.3 (5)	C6—C23—C24—C25	−177.7 (3)
C6—C5—C10—P	−95.6 (4)	C23—C24—C25—C26	−0.4 (6)
C4—C5—C10—P	79.9 (4)	C24—C25—C26—C27	0.8 (7)
O3—P—C10—C5	−33.2 (3)	C24—C25—C26—F	180.0 (4)
C11—P—C10—C5	−155.6 (3)	F—C26—C27—C28	−179.1 (4)
C17—P—C10—C5	90.5 (3)	C25—C26—C27—C28	0.1 (8)
O3—P—C11—C12	−2.7 (4)	C24—C23—C28—C27	1.8 (6)
C17—P—C11—C12	−126.4 (3)	C6—C23—C28—C27	178.6 (4)
C10—P—C11—C12	120.9 (3)	C26—C27—C28—C23	−1.4 (7)
O3—P—C11—C16	178.8 (3)		

### Hydrogen-bond geometry (Å, º)

**Table:**

*D*—H···*A*	*D*—H	H···*A*	*D*···*A*	*D*—H···*A*
C1—H1*B*···O1^i^	0.96	2.56	3.363 (5)	141
C2—H2*C*···F^ii^	0.96	2.52	3.202 (6)	128
C13—H13*A*···O2^iii^	0.93	2.53	3.350 (6)	148

Symmetry codes: (i) −*x*+5/2, *y*+1/2, −*z*+1/2; (ii) −*x*+2, −*y*, −*z*; (iii) *x*−1, *y*, *z*.

## References

[bb1] Brieden, W. & Veith, U. (2000). Eur. Patent EP1035127.

[bb2] Enraf–Nonius (1994). *CAD-4 EXPRESS* Enraf–Nonius, Delft, The Netherlands.

[bb3] Harms, K. & Wocadlo, S. (1995). *XCAD4* University of Marburg, Germany.

[bb4] North, A. C. T., Phillips, D. C. & Mathews, F. S. (1968). *Acta Cryst.* A**24**, 351–359.

[bb5] Sheldrick, G. M. (2008). *Acta Cryst.* A**64**, 112–122. 10.1107/S0108767307043930 18156677

